# Incidence of Skin Cancer in Patients With Chronic Inflammatory Cutaneous Diseases on Targeted Therapies: A Systematic Review and Meta-Analysis of Observational Studies

**DOI:** 10.3389/fonc.2021.687432

**Published:** 2021-06-03

**Authors:** Salvatore Crisafulli, Lucrezia Bertino, Andrea Fontana, Fabrizio Calapai, Ylenia Ingrasciotta, Massimiliano Berretta, Gianluca Trifirò, Claudio Guarneri

**Affiliations:** ^1^ Department of Biomedical and Dental Sciences and Morphofunctional Imaging, University of Messina, Messina, Italy; ^2^ Department of Clinical and Experimental Medicine, University of Messina, Messina, Italy; ^3^ Unit of Biostatistics, Fondazione IRCCS Casa Sollievo della Sofferenza, San Giovanni Rotondo, Italy; ^4^ Department of Chemical, Biological, Pharmaceutical and Environmental Sciences, University of Messina, Messina, Italy; ^5^ Department of Clinical and Experimental Medicine, Section of Infectious Diseases, University of Messina, Messina, Italy; ^6^ Department of Diagnostics and Public Health, University of Verona, Verona, Italy

**Keywords:** skin cancer, non-melanoma skin cancer, melanoma, biologics, psoriasis

## Abstract

Cancer is one of the several comorbidities that have been linked with chronic cutaneous inflammatory diseases namely psoriasis/psoriatic arthritis and hidradenitis suppurativa. Although the chronic inflammatory state, typical of the diseases, may induce pro-tumorigenic effects, the debate whether or not the drugs currently used in clinical practice do in facts increase a patient’s risk of malignancy remains largely unsolved. The therapeutic armamentarium has been greatly enhanced at least in the last two decades with the advent of biologics, a heterogeneous group of laboratory-engineered agents with more in the pipeline, and other targeted small molecules. Among the organ systems, skin results as one of the most commonly affected, non-melanoma skin cancers being the main drug-induced manifestations as side effect in course of these treatments. The objective of the study is to systematically review the cutaneous malignancy risk of the newer therapies through an overview of meta-analyses and observational studies on the topic.

## Introduction

Psoriasis, psoriatic arthritis and hidradenitis suppurativa are three common inflammatory and immune-mediated skin diseases characterized by increased levels of pro-inflammatory cytokines and chemokines such as tumor necrosis factor (TNF)-α, interleukin (IL)-17 and IL-23 (1–7). Chemical inflammatory mediators involved in the pathogenesis of these diseases may increase the risk of malignancies through the induction of pro-cancerous mutations, adaptive responses, resistance to apoptosis and environmental changes such as the stimulation of angiogenesis (8, 9). A number of observational studies suggested that patients affected by these diseases are at increased risk of developing cancer (10–13). In particular, increased rates of cancer, especially keratinocyte skin cancer and lymphomas were reported in patients with psoriasis or psoriatic arthritis (14). A significantly increased risk of overall cancer was observed also among patients affected by hidradenitis suppurativa in a recently published population-based cohort study (15).

The recent marketing of systemic biological (i.e. the TNF-α inhibitors etanercept, infliximab and adalimumab, the anti-IL-12/23 ustekinumab, the IL-17/IL-17 receptor antagonists secukinumab, ixekizumab and brodalumab and the anti-IL-23 agents tildrakizumab, guselkumab and risankizumab) and chemically synthetized drugs (e.g. apremilast and tofacitinib) as targeted therapies has improved the management of these diseases (16–18). However, since these drugs target molecules that may be relevant to cancer immunosurveillance mechanisms, some concerns were raised about their association with an increased risk of cancer occurrence (19–23). A recent meta-analysis of randomized clinical trials (RCTs) and open-label extension (OLE) studies reported that TNF inhibitors are associated with an increased risk of non-melanoma skin cancers (NMSC) in people with psoriasis. However, the authors of this study found that no real-world evidence was available and acknowledged the significant limitations associated with the study design of the articles included, that make it difficult to extrapolate to real-world practice (24). Evidence on the risk of skin cancer in patients with chronic inflammatory cutaneous diseases on targeted therapies is still sparse controversial. Therefore, the aim of this systematic review and meta-analysis was to assess the risk of cutaneous malignancies in patients with psoriasis, psoriatic arthritis or hidradenitis suppurativa treated with targeted therapies.

## Methods

### Search Strategy and Study Selection Criteria

This systematic review and meta-analysis was conducted in accordance with the Preferred Reporting Items for Systematic Reviews and Meta-Analyses (PRISMA) statement, following an *a priori*-established protocol registered on the International Prospective Register of Systematic Reviews (PROSPERO: CRD42020212137). The completed PRISMA checklist is provided in **Supplementary Figure 1**. Two authors (SC, FC) independently searched the bibliographic databases PubMed and EMBASE for literature related to the risk of skin cancer in patients affected by inflammatory cutaneous diseases and treated with targeted therapies. Literature was searched from databases inception until 15^th^ September 2020. The search strategy concerned terms related to inflammatory cutaneous diseases (i.e. psoriasis, psoriatic arthritis and hidradenitis suppurativa), skin cancers (e.g. squamous cell carcinoma, basal cell carcinoma and melanoma) and targeted therapies (i.e. etanercept, infliximab, adalimumab, ustekinumab, secukinumab, ixekizumab, brodalumab, tildrakizumab, guselkumab, risankizumab, apremilast and tofacitinib). Citations, titles and abstracts were exported into Endnote X9. The detailed literature search strategy for different databases is provided in **Supplementary Table 1**. Original observational studies were included if they (a) included patients affected by psoriasis, psoriatic arthritis or hidradenitis suppurativa; (b) clearly reported a well-defined measure of skin malignancies incidence; (c) included patients treated with biological drugs and/or the small molecules, apremilast and tofacitinib; (d) were written in English. To reduce the risk of publication bias, conference abstracts were also eligible for inclusion. Narrative or systematic reviews, meta-analyses, book chapters, editorials and pooled analyses were not included, but the reference lists in reviews and meta-analyses were screened to potentially identify further studies to include.

After duplicate studies were removed, two authors (SC and FC) individually reviewed titles and abstracts to remove clearly irrelevant articles and, subsequently, full text of the articles that both reviewers considered potentially eligible. Any inconsistencies were resolved at this stage through discussion or the intervention of a third independent assessor (GT or CG).

### Data Extraction

For eligible studies, information on the following items was independently collected by the same two authors and stratified by skin cancer type: study authors, year of publication, catchment area, data source, study population, study years, study design and risk estimate. Any disagreements were resolved by consensus with a third author (GT or CG).

### Assessment of Risk of Bias and Overall Quality of the Evidence

The risk of bias of the observational studies included in this systematic review was independently assessed by two authors (SC and FC) using the Newcastle-Ottawa quality assessment scale (25). This instrument consists of eight different domains for cohort studies (representativeness of the exposed cohort, selection of the non-exposed cohort, ascertainment of exposure, demonstration that outcome of interest was not present at start of study, comparability of cohorts on the basis of the design or analysis, assessment of outcome, follow-up long enough for outcomes to occur, adequacy of follow up) and case-control studies (adequate case definition, representativeness of the cases, selection of controls, definition of controls, comparability of cases and controls on the basis of the design or analysis, ascertainment of exposure, same method of ascertainment for cases and controls, non-response rate). The included studies were categorized as “low risk of bias” if at least six of the eight domains were judged to be at low risk of bias.

### Statistical Analysis

For each included study, skin cancer incidence rates (IR) per 10,000 person-years (PY) were considered as the primary outcome for the meta-analysis. Meta-analysis of IRs was performed assuming that the logarithm of each study-specific rate was normally distributed and the corresponding standard error, used to perform the inverse-variance weighting, was computed from the 95% CI (or p-value) reported in the original IRs. Between-study heterogeneity of the estimates was assessed using the Cochran’s Q-test (26) along with its derived measure of inconsistency (I^2^), and was considered to be present when Cochran’s Q-test p-value was < 0.10 or I^2^ > 40% (27). Estimates were summarized by fixed-effects or random-effects models, according to the absence or the presence of heterogeneity, respectively. It is generally accepted that when there are fewer than ten studies in a meta-analysis, both meta-regression (27) and test for publication bias (28) should not be considered. Both the study specific as well as the pooled epidemiological estimates, were graphically depicted, with their 95% CI, on a forest plot. Analyses were stratified for specific skin cancer types, i.e. NMSCs and melanoma. If a study presented more than one estimate, the most recent one was used. Two-sided p-values<0.05 were considered for statistical significance. All calculations were carried out using R Foundation for Statistical Computing (version 4.0, package: metafor).

## Results

### Characteristics of the Studies Included

**Figure 1:**
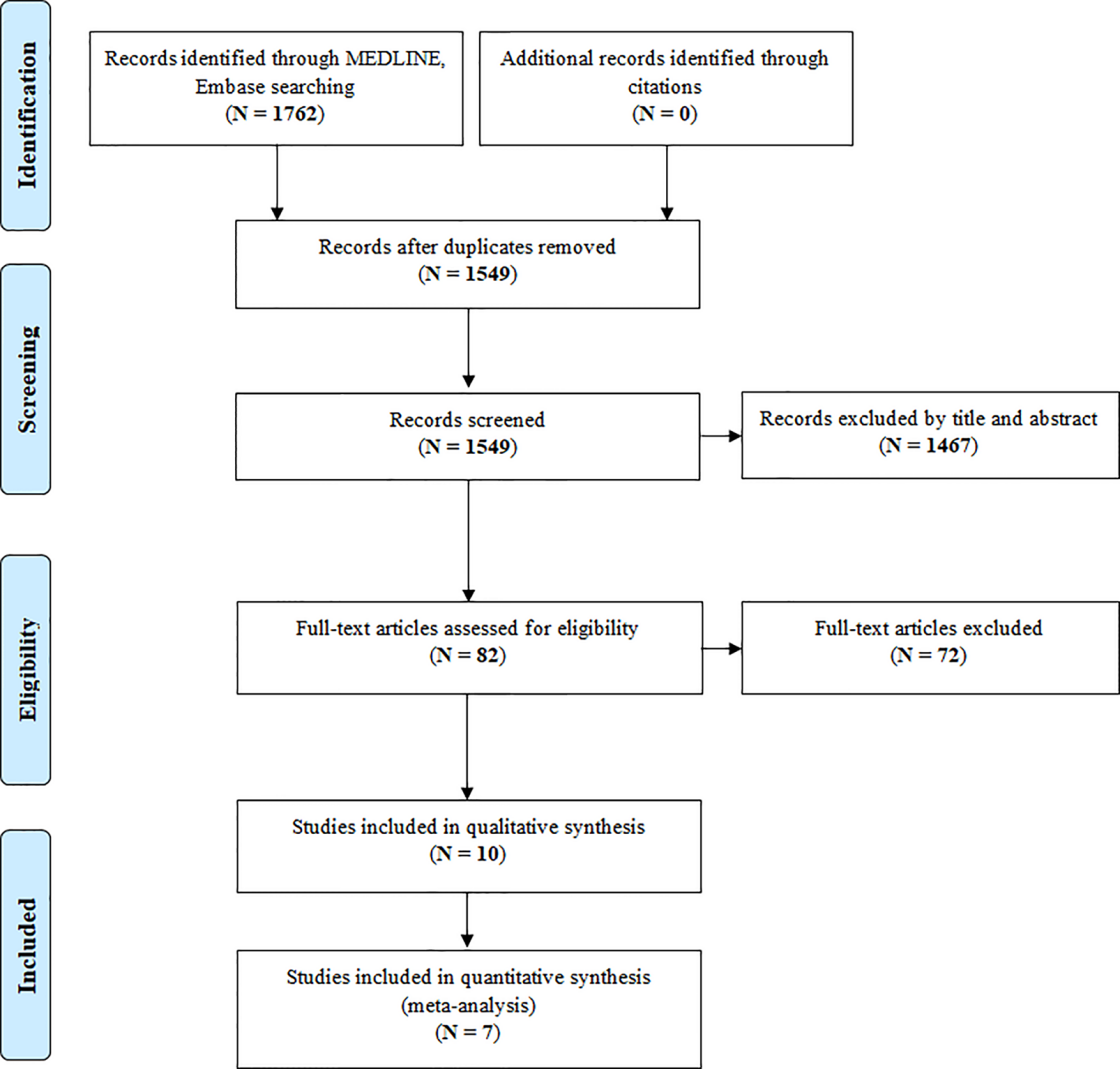
PRISMA flow-chart showing the process of literature search and study selection.

The original electronic search yielded 1762 (1549 after removing duplicates) papers potentially relevant for this review (**Figure 1**). After removing duplicates, 1549 were initially screened. Of these, 1467 were excluded after the screening of study titles and abstracts. The remaining 82 studies were retrieved for more detailed evaluation and 10 of them met the review inclusion criteria. The main characteristics of the included studies are reported in **Table 1**. Most of the included studies were prospective cohort studies (N= 5; 50.0%) (33–36, 38), three (30.0%) (29, 31, 32) were retrospective cohort studies, one was a nested case-control study (10.0%) (37) and one was a study comparing clinical trials data and real-world data (10.0%) (30).

**Table 1:** Characteristics of the studies included in the systematic review.

Reference	Catchment area	Data source	Study population	Study drugs	Study years	Study design	IR per 10,000 PYs [95%CI]
**Non-melanoma skin cancer**							
29	California (USA)	Kaiser Permanente Northern California (KPNC)	All KPNC members aged ≥ 18 years, diagnosed with psoriasis between 1998 and 2011 and treated with a systemic antipsoriatic agent	Adalimumab, etanercept, infliximab, ustekinumab	1998-2011	Retrospective cohort study	120 [98-143]
30	USA	US Truven MarketScan database	Patients with moderate to severe PsA, defined by ≥1 inpatient or ≥2 outpatient 696.0 diagnosis codes on 2 unique calendar days	Adalimumab, etanercept, infliximab, apremilast	2010-2015	Clinical trial and real-world data comparison	149.3 [116.5-182.0]
31	United Kingdom	British Society for Rheumatology Biologics Register + National cancer and death registers	All patients diagnosed with PsA starting a TNF-inhibitor and registered in the British Society for Rheumatology Biologics Register	Etanercept, adalimumab, infliximab	2002-2012	Retrospective cohort study	N.A.
32	USA	Market-Scan^®^ database and Medicare	Patients with a diagnosis of psoriasis, with the first outpatient qualifying ICD-9 CM code	Etanercept Adalimumab Infiximab	2005-2009	Retrospective cohort study	185.8 [160.2-211.42]
33	USA, Canada, Germany, France, Czech Republic, Greece, Netherlands, Spain, UK, Austria, Denmark, Ireland, Sweden	ESPRIT Registry	Patients aged ≥ 18 years of age with chronic plaque psoriasis who had been prescribed adalimumab	Adalimumab	2008-2015	Prospective cohort study	62 [52-72]
34	Canada	OBSERVE-5 surveillance registry	Adult patients with moderate to severe psoriasis initiating etanercept	Etanercept	2006-2012	Prospective cohort study	125 [60-240]
34	USA	OBSERVE-5 surveillance registry	Adult patients with moderate to severe psoriasis initiating etanercept	Etanercept	2006-2012	Prospective cohort study	262 [220-310]
35	Germany	The German Psoriasis Registry PsoBest	Adult patients with moderate-to-severe psoriasis at the time point of a new drug to be started	TNF-α inhibitors	2008-2012	Prospective cohort study	38 [12-90]
35	Germany	The German Psoriasis Registry PsoBest	Adult patients with moderate-to-severe psoriasis at the time point of a new drug to be started	Ustekinumab	2008-2012	Prospective cohort study	24 [10-136]
36	The Netherlands	Radboud University Nijmegen Medical Centre pharmacovigilance registry	Patients starting biological treatment for psoriasis in the Dermatology outpatient clinic of the Radboud University Nijmegen Medical Centre	Etanercept, adalimumab, infliximab, ustekinumab	2005-2010	Prospective cohort study	N.A.
**Melanoma**							
29	California (USA)	Kaiser Permanente Northern California (KPNC)	All KPNC members aged ≥ 18 years old, diagnosed with psoriasis between 1998 and 2011 and treated with a systemic antipsoriatic agent	Adalimumab, etanercept, infliximab, ustekinumab	1998-2011	Retrospective cohort study	8 [3-14]
31	United Kingdom	British Society for Rheumatology Biologics Register + National cancer and death registers	All patients diagnosed with PsA starting a TNF-inhibitor and registered in the British Society for Rheumatology Biologics Register	Etanercept, adalimumab, infliximab	2002-2012	Retrospective cohort study	NA
37	America and Europe	Psoriasis Longitudinal Assessment and Registry (PSOLAR)	Patients aged ≥ 18 years with moderate-to-severe psoriasis who were receiving, or were candidates to receive, systemic therapy	TNF-α inhibitors	2007-2015	Nested case-control study	NA
37	America and Europe	Psoriasis Longitudinal Assessment and Registry (PSOLAR)	Patients aged ≥ 18 years with moderate-to-severe psoriasis who were receiving, or were candidates to receive, systemic therapy	Ustekinumab	2007-2015	Nested case-control study	NA
38	USA, Canada, Germany, France, Czech Republic, Greece, Netherlands, Spain, UK, Austria, Denmark, Ireland, Sweden	ESPRIT Registry	Patients aged ≥ 18 years of age with chronic plaque psoriasis who had been prescribed adalimumab	Adalimumab	2008-2013	Prospective cohort study	5 [3-10]
35	Germany	PsoBest Registry	Adult patients with moderate-to-severe psoriasis at the time point of a new drug to be started	Adalimumab, etanercept, infliximab	2008-2012	Prospective cohort study	8 [0-43]

ICD-9 CM: international classification of diseases, 9^th^ revision, clinical modification; IR, incidence rate; NA, not available; PsA, psoriatic arthritis; PYs, person-years; SIR, standardized incidence ratio; TNF, tumor necrosis factor; UK, United Kingdom; USA, United States of America.

All included studies focused on the incidence of skin malignancies in patients treated with TNF-α inhibitors, three of them included also patients treated with ustekinumab (29, 35, 36) and only one study reported NMSC IRs also for apremilast and tofacitinib (30). No observational studies assessing the incidence of skin cancer in patients with inflammatory cutaneous diseases and treated with secukinumab, ixekizumab, brodalumab, tildrakizumab or risankizumab were found. All the included studies used real-world data sources, such as drug or disease registries and claims databases.

Of the 10 studies included in this systematic review, 7 provided data suitable for meta-analysis.

### Risk of Bias in Individual Studies

**Figure 2:**
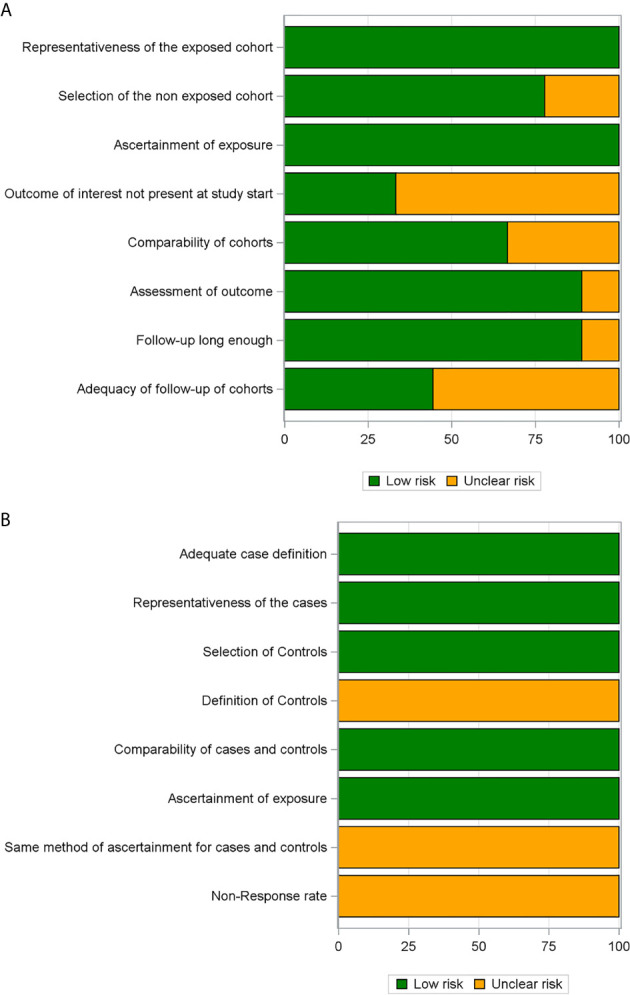
Risk of bias assessment through the Newcastle-Ottawa Scale presented as percentages across all included cohort studies **(A)** and case–control studies **(B)**.

**Figure 2** summarizes the risk of bias assessment of individual studies. The overall risk of bias was rated as low for 7 (29, 30, 32–34, 35, 38) of the 10 included studies, while 3 (31, 36, 37) studies proved to have an unclear risk of bias. Limitations mainly concerned the assessment of the presence or absence of prognostic factors and the adequacy of follow-up.

### Targeted Therapies and Skin Cancer Incidence Rates

**Figure 3:**
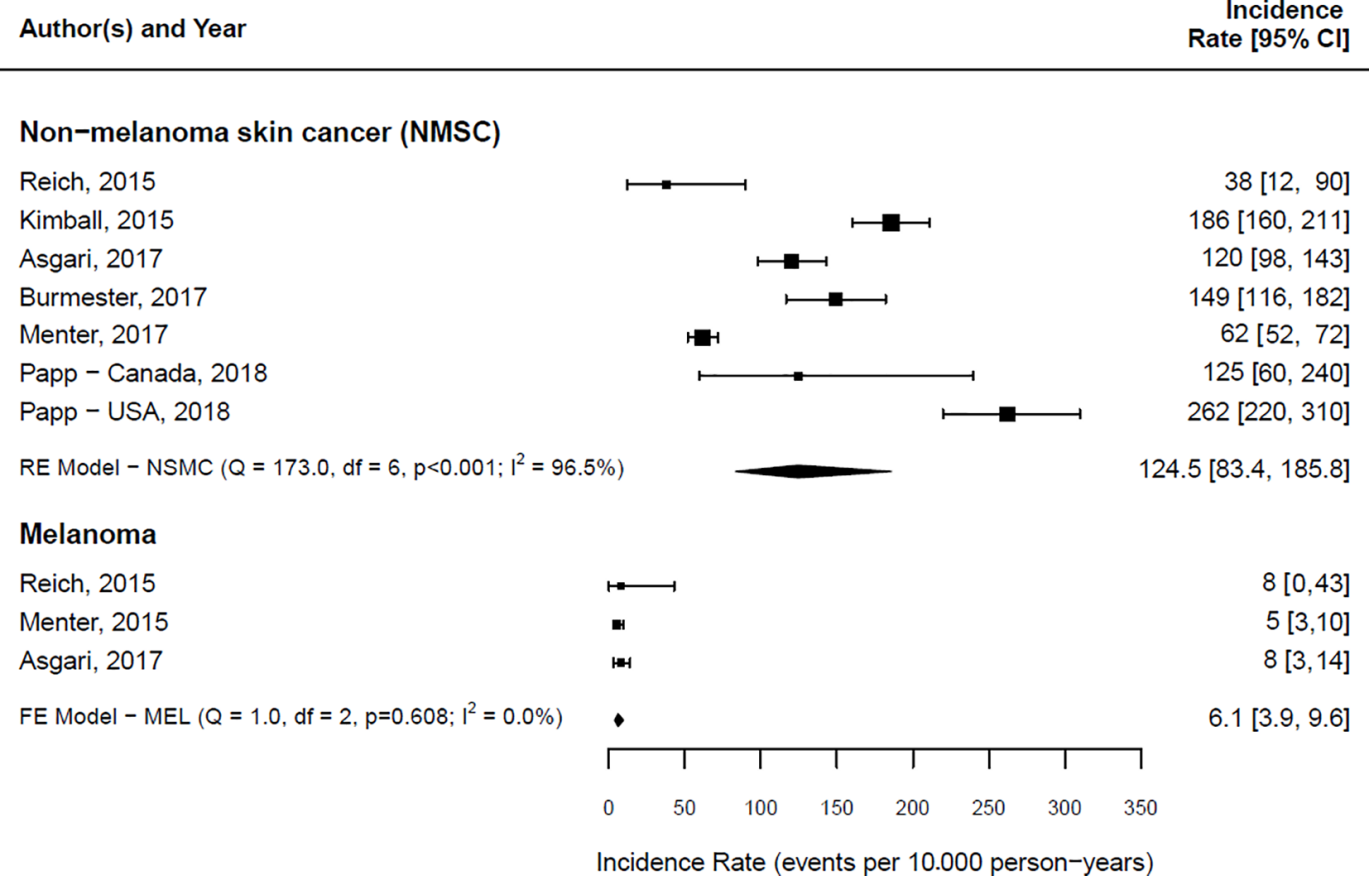
Forest plot of the estimated skin cancer incidence per 10,000 person-years along with 95% confidence intervals, stratified by skin cancer type. RE, Random-Effects model; FE, Fixed Effect model.

IRs of NMSC and melanoma reported in the articles included in this systematic review are summarized in **Figure 3**.

Overall, the IR of NMSC in the included studies ranged from 38 (95% CI: 12-90) (35) to 262 (95% CI: 220-310) (34) cases per 10,000 PYs. The pooled IR for the overall risk of NMSC was 124.5 (95% CI 83.4 – 185.8) per 10,000 PYs. A considerable heterogeneity was found among these studies (Cochrane’s Q = 173.0; I^2 =^ 96.5%).

A comparison of the incidence ratio for the overall risk of NMSC in patients exposed to biologics and small molecules versus non-biologic drugs users could be obtained only in two studies (29, 36). In one case (36), the hazard ratio (HR) was 1.42 (95% CI:1.12-1.80), while in the other one the Incidence Rate Ratio (IRR) was 0.74 (95% CI:0.60-0.91) (29).

The IR of melanoma in the included studies ranged from 5 (95% CI: 3-10) (38) to 8 (95% CI: 0-43) (35) cases per 10,000 PYs. The pooled IR for the overall risk of melanoma was 6.1 (95% CI 3.9 – 9.6) per 10,000 PYs. No heterogeneity among studies reporting melanoma IRs was found (Cochrane’s Q= 1.0; I^2 =^ 0.0%). The only study reporting an HR for melanoma between users of biologic drugs and small molecules versus non-biologic users (36) showed no statistically significant difference (HR:1.57, 95% CI: 0.61-4.09).

It was not possible to investigate both the source of heterogeneity and the presence of publication bias, as fewer than ten studies were included in the meta-analysis (28).

## Discussion

In recent years, we have witnessed a revolution in the treatment of many skin diseases, ranging from bullous diseases, urticaria, atopic dermatitis, to hidradenitis suppurativa and psoriasis (39). In particular, psoriasis is a chronic cutaneous inflammatory disease affecting an estimated 125 million people worldwide, that is often associated with systemic manifestations such as major adverse cardiovascular event, obesity, inflammatory bowel disease and arthropathic psoriasis (40, 41). The decision to use one therapy over another is significantly influenced by these comorbidities and the severity of the disease. Moreover, a better understanding of the pathogenesis of this systemic disease had led to identification of new therapeutic targets (42). Whereas the older treatment options, such as phototherapy, methotrexate and cyclosporine A, are still effective, biotechnological drugs are substantially improving the therapeutic arsenal. The success of these new therapies lies in their great selectivity of action which allows to obtain, in most cases, a significant therapeutic efficacy in a short time with a reduction in side effects compared to traditional therapies. Through these therapies, even the severest symptoms of psoriasis and psoriatic arthritis can be excellently treated (43, 44). The biological drugs produced so far are monoclonal antibodies and fusion proteins. These products have the enormous advantage of being able to selectively interfere, at various levels and with different modes of action, in the immunological processes that trigger and sustain psoriasis (45). To date they are divided into five classes: TNF-α inhibitors, IL-12/23 inhibitors, IL-17 inhibitors, IL-23 inhibitors and phosphodiesterase type 4 (PDE4) inhibitors (40).

According with the above-mentioned results, our review found no observational studies assessing the incidence of skin cancer in patients with inflammatory cutaneous diseases and treated with biologics targeting selectively IL-17 or IL-23, thus obtaining mainly data on patients under anti-TNF-α therapy and, to a more limited degree, under ustekinumab, apremilast and tofacitinib.

TNF-α inhibitors infliximab, etanercept, adalimumab and certolizumab pegol are the oldest class of currently approved biotechnological drugs for the treatment of both psoriasis and psoriatic arthritis and, limited to adalimumab, of hidradenitis suppurativa. TNF-α exerts several effects. It could promote the progression of cancer (46), but also blocking TNF-α could result in arresting antitumor immune response and in promoting the growth of immunogenic tumors (47–49).

Some of the studies analyzed in this systematic review also included patients receiving ustekinumab, apremilast and tofacitinib (29, 30, 35, 36). Ustekinumab belongs to the class of biologics targeting the IL-12/23 pathway, whereas apremilast is an anti-PDE4 small molecule and tofacitinib a janus kinase inhibitor. The inhibition of these pathways causes a downregulation of the inflammatory response by modulating the expression of TNF-α, IL-23, IL-17 and other inflammatory cytokines, all involved at least in part in the tumorigenesis.

Consequently, whereas these drugs have shown dramatically excellent efficacy, concerns have been raised about the risks related to this class of agents.

Undoubtedly, patients with psoriasis are at an increased risk of cancer. Assessing the baseline risk of cutaneous malignancies in psoriasis patients is challenging due to most studies including both treated and untreated patients, and due to confounding factors like phototherapy and immunosuppressive therapy (50). Moreover NMSC and melanoma are known to arise with increased incidence among patients that have undergone medical radiation procedures or immunosuppressive therapy (51–53), such as those immunosuppressed in an iatrogenic way after a solid organ transplantation (54–56). According to the World Health Organization, age standardized world incidence of melanoma and NMSC are respectively 3,4 and 11 per 100.000 PYs. On the other hand, recent data emerging from literature show that skin cancers have a higher incidence in psoriasis patients than general population with a standardized incidence ratio of 3.37 (95% CI 1.84-5.66) (57). More in detail, Pouplard et al. in a meta-analysis reported a standardized incidence ratio of 5.3 for squamous cell carcinoma (SCC) (95% CI 2.63–10.71) and of 2.00 for basal cell carcinoma (BCC) (95% CI 1.83–2.20), whereas the authors reported a similar risk of melanoma in psoriatic patients compared to the general populations.

When considering the risk of skin cancer in psoriatic patients under treatment, many aspects should be analyzed: predisposing factors, duration and timing of exposure, the cumulative dose, the interaction with other carcinogens and, also, the latency. Despite all these data to be considered, enough evidence confirmed the relation between skin cancer and specific treatment for psoriasis and it has emerged that the risk increases even more respect untreated patients (58).

In particular, oral psoralen and ultraviolet A (PUVA) is associated with an increased risk for skin cancer in a dose dependent fashion: risk of NMSC is greatest with >350 treatments, while melanoma risk is increased with >250 treatments (59, 60). However, the carcinogenic mechanism of PUVA has not been elucidated: it maybe acts in a mutagenic and immunologic way (61). Instead, even if UVB phototherapy may increase photoaging acting with multiple mechanisms (inhibition of DNA synthesis, epidermal keratinocyte hyperproliferation, induction of T-cell apoptosis and of anti-inflammatory cytokines), no increase in skin cancer has been observed, especially with <100 treatments. Only when patients have been treated previously with PUVA and, in a second time, with broadband UVB (>300 treatments), it has been noted a modest increase in SCC (incidence rate ratio 1.37, 95% CI 1.03–1.83) and BCC (incidence rate ratio 1.45, 95% CI 1.07–1.96) (62).

Also systemic non biologic therapies are associated with an increased risk of skin cancers (63), acting primarily as immunosuppressants. Treatment with methotrexate results in higher risk for NMSC, but no association with risk for melanoma was observed (64). In detail, it has been shown that patients in treatment with methotrexate seem to have a doubled risk of SCC compared with people who receive PUVA therapy (65). Cyclosporine is associated with an elevated risk of SCC, which could increase even more in relation to treatment duration (>2 years) and previous therapy (PUVA) (66, 67), as already seen in transplant patients treated with high doses of cyclosporine and for long periods (68–70).

In our systematic review, we also considered studies evaluating the risk of skin cancers in patients with hidradenitis suppurativa in treatment with adalimumab, the only approved biologic agent for moderate-to-severe hidradenitis (71, 72). No articles were found that met the inclusion criteria. Nevertheless, data from literature point to a higher risk of developing NMSC in patients with hidradenitis than general population (15). Compared with psoriatic patients who underwent biologic treatment, patients with hidradenitis start treatment with TNF-α inhibitors after fewer months/years from the diagnosis of the disease and the guidelines do not provide obligatory treatment with first line systemic immunosuppressive drug, such as cyclosporine or methotrexate, before approaching the biologic therapy.

Considering all together the studies included in the metanalysis, the IR emerging from our systematic review shows an incidence of skin cancer in biologic treated patients, 124.5 per 10000 PYs for NMSC and 6.1 per 10000 PYs for melanoma. With regard to NMSC, IRs in literature presented large variability, from 24 in a psoriatic cohort of a German registry to 262 coming from a USA surveillance registry on patients treated with etanercept. The IR has been established on 8 out of 10 studies (**Table 1**). Concerning melanoma, 3 out of 6 studies reported an IR, ranging from 5 to 8 (**Table 1**). As a comparison, these IRs are significantly lower than post-transplant skin cancer IR, that is 1355 per 100.000 PYs for SCC and 125 per 100.000 PYs for melanoma (73).

Our figures substantially agree with those reported in a recent systematic review and metanalysis by Vaengebjerg et al. (14) who reviewed 112 observational studies and more than 2 million persons, thus assessing them for prevalence, incidence and overall risk of cancer in patients with psoriasis and psoriatic arthritis. The reported IR per 1000 PY for overall cancer was 11.75 (95% CI, 8.66-15.31) and 4.35 (95% CI, 3.18-5.70) for keratinocyte cancer, whereas the IR for melanoma was 0.37 per 1000 PYs.

A study by Esse and collaborators was focused on melanoma risk in patients treated with biologics for common inflammatory diseases, such as inflammatory bowel diseases, rheumatoid arthritis and psoriasis (68). In detail, they considered a total of 7 studies, consisting of patients treated with TNF-α inhibitors, one of which regarding patients with psoriasis and, moreover, included in our review (74). According with their findings, the risk of melanoma in biologic-treated patients with IBD and psoriasis compared with their biologic-naïve counterparts receiving conventional systemic therapy showed no statistically significant increases. Esse et al. included in their paper only one study (36) concerning psoriatic patients; this study is currently the only one reporting an HR for melanoma in patients treated with TNF-α inhibitors compared with non-biologic users and shows no significant difference between the two groups.

With regard to NMSC, the paper by Asgari (36) explicitly reported an HR for the same comparison. Our review considered an additional study in which we were able to calculate IRR from the reported data (29). While Asgari et al. (36) reported an increased HR for NMSC in patients treated with TNF-α inhibitors compared with non-biologic users, data coming from the other study (29) showed no statistically significant differences.

The main strengths of our analysis included the use of a well-defined protocol with strict inclusion and exclusion criteria. Complying with the protocol, our search addressed a clearly focused question with standardized data extraction and quality assessment to minimize errors. In addition, the real-world setting of the studies, the inclusion of biologic agents and of patients treated exclusively for common cutaneous inflammatory diseases represent distinctive features of our review and metanalysis.

The main limitation was the small number of eligible studies. The studies were also heterogeneous, which makes comparison difficult. In addition, a major weakness of the analysis was the absence of adjustment for established risk factors for NMSC and melanoma.

Furthermore, in previous studies performed only on patients with PSO it was found that there were no univocal data on the higher or lower incidence of tumors in patients with PSO. In particular, they were studies that analyzed both patients treated with systemic drugs and patients treated with biological drugs (50, 75). In our systematic review and meta-analysis, we considered only patients treated with target therapies suffering from psoriasis, PSA and/or HS.

In common with previous studies, on the other hand, there is the fact that the risk of skin tumors itself cannot be excluded because patients had to undergo immunosuppressive therapy (systemic or not) before being able to carry out treatment with a target therapy.

Another limit that emerges from our systematic review, in common with other articles already present in the literature, is the follow-up time. As demonstrated by many studies, the development and growth times of skin tumors are long and may exceed the observation periods of the clinical trials in the literature.

## Summary and Perspectives

Although with some limitations, the metanalysis of currently available real-world data seems to suggest that treatment of psoriasis, psoriatic arthritis and/or hidradenitis suppurativa with TNF-α inhibitors, ustekinumab, apremilast or tofacitinib does not increase the risk of NMSC or melanoma compared to “non-biologic” systemic treatments. The cumulative sample size of the studies in literature is certainly conspicuous, but, in the light of the worldwide diffusion and frequency of the aforementioned diseases as well as their multifactorial nature and response to treatment, including undesired effects, further data are desirable.

Additionally, the ending years of the periods analyzed in the available studies range from 2009 to 2015. Similar evaluations of real-world evidence concerning molecules marketed in the last 10-15 years, such as secukinumab, ixekizumab, brodalumab, tildrakizumab or risankizumab, would be of great interest, particularly when considering that these molecules are widely used in current clinical practice. Consequently, to conduct future trials it is necessary to consider the above data and the fact that the number of studies comparing newer molecules and conventional drugs are small. A greater number of new trials will have to be conducted, considering longer follow-up times and, above all, common methods will have to be applied to allow a comparison between the various studies.

In summary, this updated systematic review and meta-analysis seems to suggest that no differences exist between treatment of chronic cutaneous diseases with biotechnological drugs/small molecules and conventional DMARDs in terms of HR/IRR for melanoma, while data on NMSC are more controversial. Nevertheless, periodic dermatologic screening should be ensured for all patients undergoing these therapies.

## Data Availability Statement

The original contributions presented in the study are included in the article/**Supplementary Material**. Further inquiries can be directed to the corresponding author.

## Author Contributions

Conceptualization: CG and SC. Methodology: SC, FC, GT, LB, and CG. Validation: SC, LB, and CG. Resources: SC, AF, FC, GT, YI, LB, MB, and CG. Data curation: SC, AF, FC, YI, GT, MB, and CG. Writing-original draft preparation: SC, LB, and CG. Writing-review and editing: SC, LB, and CG. Supervision: SC, LB, and CG. All authors contributed to the article and approved the submitted version.

## Conflict of Interest

The authors declare that the research was conducted in the absence of any commercial or financial relationships that could be construed as a potential conflict of interest.
